# Draft genome of *Corynebacterium glycinophilum* S209 isolated from healthy human skin

**DOI:** 10.1128/mra.00809-25

**Published:** 2025-10-21

**Authors:** Sandra Jablonska, Lila Nelson, Grace Finger, Alex Kula, Catherine Putonti

**Affiliations:** 1 Bioinformatics Program, Loyola University Chicago 2456, Chicago, Illinois, USA; 2 Biology Department, Loyola University Chicago 224010, Chicago, Illinois, USA; Wellesley College, Wellesley, Massachusetts, USA

**Keywords:** *Corynebacterium glycinophilum*, skin microbiota

## Abstract

*Corynebacterium glycinophilum* is an understudied species of this genus, although it has been identified in dairy products as well as the human skin microbiome. Recently, we isolated a strain from the skin of a healthy human female, and here, we present the draft genome of this isolate, *C. glycinophilum* S209.

## ANNOUNCEMENT

*Corynebacterium glycinophilum* is a species first proposed in 2015, describing an isolate from a putrefied banana (1). It has subsequently been identified in metagenome-assembled genomes (MAGs) from ripening cheese (*C. glyciniphilum* isolate 122_S29 and *C. glyciniphilum* isolate 35_S49 [Accession nos. JAKEBA01000000 and JAKDKC01000000, respectively]) and a human skin sample (*C. glyciniphilum* isolate oOBflCGst5_bin.2.MAG [Accession no. CALTYC010000000]). Recently, we isolated a strain of *C. glycinophilum*, taxonomically identified via whole genome sequencing, from a skin swab.

Participants in our institutional review board (IRB)-approved study (IRB no. 3603) were students at Loyola University Chicago and self-identified as female, “healthy,” and had not taken antibiotics within the last 6 months. Each participant was instructed to rub a provided cotton swab (BD BBL CultureSwab) on the middle of their forehead for 60 seconds, after which the swab was inserted into the provided storage solution (Amies media). Within 1 hour of sampling, swabs were swirled and squeezed in the storage media and then spread on mannitol salt (MS) agar plates and incubated for 24 hours at 35°C with 5% CO_2_. Morphologically distinct colonies were selected, placed in MS broth, and incubated under the same conditions. This process was repeated to purify the isolate described here. DNA was extracted using the DNeasy Blood and Tissue kit (Qiagen), following the manufacturer’s protocol for Gram-positive bacteria. Library construction was performed by SeqCoast (Portsmouth, NH, USA) using the Illumina DNA Prep tagmentation kit and unique dual indexes and then sequenced on the Illumina NextSeq2000 platform using a 300-cycle flow cell kit (2 × 150 bp reads). Read demultiplexing, read trimming, and run analytics were performed using DRAGEN v3.10.12 (Illumina) prior to delivery from SeqCoast.

Sequencing produced 9,395,192 raw reads which were then processed and assembled using the BV-BRC v3.51.7 web tool with the “auto” option (2). First, raw reads were trimmed using trim_galore v0.6.5dev (https://github.com/FelixKrueger/TrimGalore), normalized via bbnorm v37.60 (https://sourceforge.net/projects/bbmap/), and assembled using Unicycler (v0.4.8) (3), followed by two rounds of polishing with Pilon v1.24 (4). The publicly available genome sequence was annotated via the National Center for Biotechnology Information (NCBI) Prokaryotic Genome Annotation Pipeline v6.10 (5). Genome statistics were calculated by BV-BRC. Taxonomic identification was informed by BV-BRC via taxonomic binning of the raw reads and confirmed by the JSpeciesWS (6), calculating the average nucleotide identity (ANI) between the draft genome and the sole representative of the species in the tool’s database, *C. glycinophilum* AJ 3710 (GCF_000626675.1). Completeness and contamination metrics were computed using CheckM v1.2.3 (7) upon submission to NCBI. Unless otherwise noted, default parameters were used for all software tools.

Table 1 lists the assembly statistics for the genome. Strain S209 shared 98.10% ANI with *C. glycinophilum* AJ 3710. While several *Corynebacterium* species are abundantly found on facial skin (8), further investigation is needed to ascertain if *C. glycinophilum* is a resident or transient member of this environment.

**TABLE 1:** Genome assembly statistics for *C. glycinophilum* S209

Genome Attribute	*C. glycinophilum* S209
Assembly length (bp)	3,609,534
No. of contigs	28
Contigs N50 (bp)	239,860
Coverage (x)	227.95
G + C (%)	64.6
# genes	3,416
# tRNAs	51
Completeness (%)	99.28
Contamination (%)	4.32

## Data Availability

This Whole Genome Shotgun project has been deposited in GenBank under the accession no. JBPFAP00000000. The version described in this paper is the first version. Raw sequencing data has been deposited in NCBI’s SRA database, accession no. SRR34298189.

## References

[1] Al-Dilaimi A, Bednarz H, Lömker A, Niehaus K, Kalinowski J, Rückert C. 2015. Revisiting Corynebacterium glyciniphilum (ex Kubota et al., 1972) sp. nov., nom. rev., isolated from putrefied banana. Int J Syst Evol Microbiol 65:177–182. doi:10.1099/ijs.0.065102-0 25323597

[2] Olson RD, Assaf R, Brettin T, Conrad N, Cucinell C, Davis JJ, Dempsey DM, Dickerman A, Dietrich EM, Kenyon RW, et al.. 2023. Introducing the bacterial and viral bioinformatics resource center (BV-BRC): a resource combining PATRIC, IRD and ViPR. Nucleic Acids Res 51:D678–D689. doi:10.1093/nar/gkac1003 36350631 PMC9825582

[3] Wick RR, Judd LM, Gorrie CL, Holt KE. 2017. Unicycler: resolving bacterial genome assemblies from short and long sequencing reads. PLoS Comput Biol 13:e1005595. doi:10.1371/journal.pcbi.1005595 28594827 PMC5481147

[4] Walker BJ, Abeel T, Shea T, Priest M, Abouelliel A, Sakthikumar S, Cuomo CA, Zeng Q, Wortman J, Young SK, Earl AM. 2014. Pilon: an integrated tool for comprehensive microbial variant detection and genome assembly improvement. PLoS One 9:e112963. doi:10.1371/journal.pone.0112963 25409509 PMC4237348

[5] Tatusova T, DiCuccio M, Badretdin A, Chetvernin V, Nawrocki EP, Zaslavsky L, Lomsadze A, Pruitt KD, Borodovsky M, Ostell J. 2016. NCBI prokaryotic genome annotation pipeline. Nucleic Acids Res 44:6614–6624. doi:10.1093/nar/gkw569 27342282 PMC5001611

[6] Richter M, Rosselló-Móra R, Oliver Glöckner F, Peplies J. 2016. JSpeciesWS: a web server for prokaryotic species circumscription based on pairwise genome comparison. Bioinformatics 32:929–931. doi:10.1093/bioinformatics/btv681 26576653 PMC5939971

[7] Parks DH, Imelfort M, Skennerton CT, Hugenholtz P, Tyson GW. 2015. CheckM: assessing the quality of microbial genomes recovered from isolates, single cells, and metagenomes. Genome Res 25:1043–1055. doi:10.1101/gr.186072.114 25977477 PMC4484387

[8] Jensen MG, Svraka L, Baez E, Lund M, Poehlein A, Brüggemann H. 2023. Species- and strain-level diversity of Corynebacteria isolated from human facial skin. BMC Microbiol 23:366. doi:10.1186/s12866-023-03129-9 38017392 PMC10683109

